# Supplementary material for: Dual Mechanisms of Salinity Tolerance in Wheat Germplasm Lines W4909 and W4910
Source: Int J Mol Sci. 2024 Nov 30;25(23):12892. doi: 10.3390/ijms252312892 (PMC11640813; doi:10.3390/ijms252312892)
Supplement: Supplementary file 1 [file ijms-25-12892-s001.zip › Table S3 Survival, leaf sodium concentration, and marker profiles of salt treated parental lines.pdf]

Table S3. Survival rate, survival days since treatment (SDST), leaf sodium concentration, and STS marker profiles in salt-treated parental lines.

ID # [1]	Kind	No. plants	Survival (%)	SDST	Leaf sodium conc. [2]	psr1205	M264410	M6805321
6687	Chinese Spring	8	87.5	51.88	6262	-	-	-
6598	AJDAj5	7	100	>52	4801	-	-	-
6621	Ph-Inhibitor	8	100	>52	13016	+	+	+
7151	progeny of W4909	8	100	>52	11943	+	-	-
7157	Progeny of W4910	9	100	>52	12030	-	+	+
5722	Anza	9	66.7	51.11	6610	-	-	-
5754	Yecora Rojo	9	55.6	51.45	5793	-	+	-

[1] ID number is the seed packet number, each contains seed harvested from a single plant.

[2] Mean of three samples, sodium concentration in mg Kg^{-1} DW of leaf.
